# Observations on Functional Affections of the Spinal Cord and Ganglionic System of Nerves

**Published:** 1835-01-01

**Authors:** 


### MEDICAL QUARTERLY REVIEW.

### REVIEWS.

*Observations on Functional Affections of the Spinal Cord and Ganglionic System of Nerves.* By William Griffin, m.d., and Daniel Griffin, m.r.c.s.—London, 1834. 8vo. pp. 247.

Among the papers which appeared from time to time in our excellent predecessor, the London Medical and Physical Journal, there were few more calculated to excite attention, or repay perusal, than those of Dr. Griffin, on the Functional Affections of the Spinal Cord. There was one point in those essays which we never lost sight of in our subsequent practice, namely, that in hysteria there is a tender spot in the spine, and counter-irritation directed thither will often act like a charm, and dispel at once whole trains of morbid action. This axiom has been the lode-star which has guided us in many a dark and dubious case; and it was therefore with singular pleasure that we heard that these papers, collected and continued, now formed a volume, and that we were thus about to reap the fruits of several additional years' experience. The first of these essays appeared in 1829; a fact which we merely mention, lest some one should accuse the authors before us of copying books of which their own writings were the archetype.

*Introductory Observations* form the subject of Chapter 1st; and the very first ones seem to us so just in substance, as well as so pleasing in manner, that we cannot refrain from transferring them to our pages.

"Although the attention of the medical profession has been, within these few years, so very much directed to affections of the spinal cord, it would seem, on inquiry, that little really new has been added to our previous acquisitions on the subject. Referring to many valuable opinions of German and French writers, fallen into undeserved neglect, and comparing them with inferences now almost obtruding themselves on general notice, we shall be apt to

Jan. 1835.

imagine there has been, in this country at least, an unaccountable carelessness of observation; or be led to very unsatisfactory reflections on the impenetrable obscurity in which all disorders of the nervous system would seem to be involved. Perhaps it is because of the little fruit gathered by long toil, that some are inclined to consider the labour misapplied; and while they are satisfied to treat, under the appellation of nervous or hysterical, whole trains of complaints which have little in common but that of their being ill understood, venture to censure the persevering efforts of those, who, if they have been as unsuccessful, have at least the merit of not being so hopeless, in the inquiry. It is gratifying to observe that the rapid progress which modern physiology is making, daily exposes the futility of opinions which measure the value of application only by its ill success in particular instances, and offers new proof that discoveries in obscure sciences depend almost as much on the expenditure of time and thought in their pursuit, as on the strength of the intellect which may make them.

"But, exclusive of these considerations, and even of the temptation to new exertion in the late extraordinary discoveries elicited by the labours of Magendie, Le Gallois, Wilson Philip, and Charles Bell, the subject of spinal disease holds out a most imperative inducement, in the perplexed state of our diagnosis through a vast range of complaints, and in the acknowledged mistakes which we every day see made in the practice of well-informed members of the profession. We need only refer to the periodicals of the day, which teem with cases of, as they are called, strange, anomalous, proteiform maladies, the mocking-birds of nosology, or imitators of every known disease, accompanied with cautions on the danger of confounding them with their prototypes: or to the admitted difficulty in the treatises on nervous and hysteric diseases by the most distinguished practitioners of the day, of offering any marked symptoms by which they could always be clearly distinguished.* Indeed, the actual existence of these singular and apparently func-

"* Dr. Hamilton, in his Treatise on Diseases of Women and Children, says, 'On some occasions, hysterics put on the appearance of several disorders, such as melancholy, epilepsy, palsy, inflammation of the lungs or bowels, gravel, &c. It requires in these cases not only the *most unremitting attention*, but also the *utmost practical discernment*, to distinguish the true disease from that which it resembles.

'When the symptoms are not uniformly and regularly those which occur in the ordinary cases of the disease imitated; when there suddenly seems great danger, without those previous changes in the progress of the complaint which are usually met with; when there is either a natural state of the pulse, with alarming symptoms, or a very frequent irregular pulse, without any affection of the breathing or shrinking of the features, there is reason to suspect hysterics as the true disorder. *Cases from time to time occur where it is impossible to ascertain the real nature of the affection, till towards its termination.* The fact, too, that in every acute disease of women which requires copious evacuations, or which debilitates the system, hysterics are apt to occur in the progress to recovery, adds much to the difficulty of judging precisely in any given case.'

"Dr. Gregory, in his Practice of Physic, after mentioning the frequent resemblance and connexion between hysteria and epilepsy, and the difficulty of tional disorders, which assume the symptoms of all others, and continue unrelieved by the remedies applicable to any; which look like inflammations, and are rendered worse by bloodletting; or simulate intense spasmodic attacks, and bid defiance to opiates; and which yet, after resisting all possible treatment, eventually, and perhaps suddenly, disappear of themselves; would, in a merely therapeutic view, appear strange, and deserving of inquiry.

"In the classification of diseases, the great division into those of the vascular and nervous system, the pyrexiæ and neuroses of Cullen, was at once obvious to the nosologist; and again, the distinction of nervous complaints, as they developed themselves in any one tissue or organ, or another, and invariably presented the same characters, seemed easy of attainment; but there came to be a great difficulty about that vast number of the latter which are so variable in their seat and appearance, and so untraceable in their origin, as apparently to preclude all proper arrangement. They were necessarily thrown together as a sort of anomalous class, like the Cryptogamia in botany, awaiting the result of future discovery for more appropriate distribution. But surely, if it had been considered that all nervous disorders must eventually resolve themselves into affections of the three great centres of nervous influence, the cerebral, the spinal, and ganglionic masses, much of this perplexity might have been avoided. We have, it may be presumed, now made sufficient progress in the pathology and diagnosis of diseases of the brain to estimate with some accuracy the characters which indicate their origin, and may generally, even in those that most nearly resemble spinal or other affections, draw correct inferences from the history of the case and the intensity of particular symptoms. Of those of which there is much doubt, or which evidently do not belong to the brain, we can observe how far they correspond with the usual or known symptoms of spinal disorder, organic or functional; and, if there be some found bearing no analogy to either the one or the other, the deduction seems clear, that they depend on some disordered or diseased state of the ganglionic system. However hypothetical a division of this kind may appear in diseases of the nervous tissues, which are so delusive and difficult of arrangement, and after death present so few appearances to direct the reasonings of the pathologist; however frequently erroneous its application may prove, it must be true in principle, and, when once held in view, cannot but give more of method and object to our investigations, and greater rationality to our treatment." (P. 1.)

distinguishing one from the other, says, 'But it is not only from epilepsy that hysteria is difficultly distinguished. There is hardly a disease in the whole nosology, of which it has not imitated the symptoms, and that with surprising accuracy. I have seen hysteria accompanied by constant vomiting; by a complete ischuria renalis; by the most obstinate colic; by all the symptoms of genuine asthma. Authors have described in like manner a hysterical jaundice, a hysterical mania, a hysterical diabetes. These circumstances require to be borne in mind with reference to prognosis.' "

Our authors observe, that when a subject is universally admitted to be perplexing, any name or phrase is accepted, to relieve us from the imputation of total ignorance. "To this alone can be attributed the ready acceptance of the words imitative, proteian [Protean], anomalous, &c., as applied to hysteric and nervous diseases, as if there existed in the animal economy some evil influence, without home or habit, or relation, capable of increasing or interrupting any of its functions, or assuming any of its morbid actions, yet free and independent of all organic change. The convenience of referring to such influence all the morbid phenomena which are difficult of explanation, bears a just proportion to its mysterious nature: but surely we might as well speak of labour imitating cramp, as of hysteria imitating croup,—the one a spasmodic affection of the gastrocnemii muscles, occasioned by pressure or irritation of the sacral nerves; the other a spasmodic affection of the muscles of the larynx, occasioned by irritation of the cervical." (P. 5.)

The comparison at the end of the passage just quoted is more sprightly than accurate; for we may suppose the spasm of the gastrocnemii to be identical, whether produced by pressure on the sacral nerves during parturition, or by any other cause; whereas, in the case of hysteria imitating croup, there is an essential difference between the original and the copy; for in croup there is a real inflammation and a false membrane as its product; but hysteria, however stridulous may be the voice, however *croupy* the breathing, leaves no such wreck behind. Yet the subsequent question is just and ingenious. "If we were to inquire, in a case of labour, what is this spasmodic and painful affection of the gastrocnemii? and it was answered, It is not idiopathic cramp, but an affection exactly resembling it, dependent on the parturient state,—one, in fact, of the many anomalous complaints which labour is found to imitate,—would it be conceived in the slightest degree satisfactory?" (P. 6, *note.)*

The following remarkable case, which is very interesting in itself, becomes doubly so to every reader of this work, from its having been the first which especially directed the attention of our authors to the disorders of the nervous system.

"A young lady, aged twenty-one, who had always before enjoyed good health, received a slight blow on the chest from her mother, during her convulsive struggles while dying of apoplexy. She spit up a little blood at the time, and felt pain for some days: after this it suddenly removed to the abdomen, affecting the left side, about the situation of the descending colon, and was accompanied by frequent pulse, tenderness, and the most incessant vomiting. The pain was abated by bleeding, blistering, and aperients; but nothing could allay the vomiting, which was brought on by the smallest quantity of any thing, solid or liquid, taken into the stomach. This came to be attended with flitting pains in the head, with throbbing of the temples, and intolerance of light, attributed to the straining, the continuance of which made it difficult to move the bowels. Even when medicine did operate, it gave no relief.

"She remained many days in this state, suffering much from want of rest and the distressing retching; after which she was attacked with frequent oppression, occurring at intervals through the day, and usually terminating in fits of insensibility. In these she usually lay for ten or fifteen minutes, with her hands fast clenched, or sometimes shutting and opening them alternately with great rapidity. There was considerable rigidity of the tendons of the wrist, while the fit lasted; and the first symptom of amendment was always a gradual relaxation and opening of the fingers, when she fetched a long deep sigh, and recovered.

"These oppressions proved as intractable as the vomiting, and were very distressing. Repeated blistering, ether, assafœtida, opium, and other antispasmodics, were had recourse to without relief, except of the most temporary kind. At the end of three weeks, however, the more severe symptoms of the complaint, without any very obvious cause, and after resisting every kind of treatment, began gradually to decline: the oppressions, throbbing at the temples, fits of insensibility and vomiting, manifestly abated; and the digestive organs, the state of which had never been lost sight of, improved rapidly under mild aperients and bitters. In short, she soon after recovered a sufficient degree of health to permit her going to a party, and even joining in the amusements.

"This reprieve was but of very short continuance. A return of the oppression brought with it cough, pain in the chest and left side; the former slowly disappearing as the latter symptoms advanced and became more formidable. The cough was loud, dry, and convulsive, and became at last so incessant, that she had no intermission of the fits by day or by night. The convulsive expirations followed one another with such rapidity, that one could only conceive the suffering by imagining the fits of a severe chincough following one another without interval. To heighten the distress, it increased considerably the pain in the chest and sides, and the respiratory muscles became so sore and tender, from the eternal convulsive action, that she could scarcely bear to have a finger touch them. After much time had passed in vain attempts to remove or alleviate it, she became affected with swelling and pain in the anterior part of the right lobe of the liver, which increased rapidly, and formed a round, circumscribed, shining tumor, bearing all the appearance of an abscess. This was very painful, and the torture produced by the constant coughing was extreme." (P. 7.)

A consultation was now held, and blue pill prescribed. Profuse salivation was produced, and the patient experienced temporary relief; but new symptoms continually supervened, and the case was considered hopeless. But a correct diagnosis was at last made.

"On an accidental visit of her medical attendant at the close of the year 1828, the connexion between several of the pains of which she complained and the distribution of the spinal nerves appeared so striking, that an examination of the spine was made. There was no deformity, unevenness, or prominence of the vertebræ, but extreme tenderness of the whole column. Pressure on any of the spinous processes excited instant convulsive fits of coughing, and pain at the corresponding point anteriorly, or oppression. The slightest curvature in any direction was intensely painful; attempting to turn in the bed during the examination (which, however, she could never either accomplish or permit,) occasioned a sensation as if her back was breaking; raising the head from the pillow, and bending the neck forward, brought on a burning pain at the middle dorsal vertebræ, which shot down to the extremity of the spine, and thence to the limbs, knees, and toes, followed by a sort of general cramp. It seemed extraordinary how little the patient directed attention to the back in so intense a case of spinal disease: she frequently complained of pain there; but, as it was never constant, like those felt at the extremities of the nerves, and was only excited by pressure or motion of the spine, and was then generally accompanied by, or occasioned, extreme sickness of stomach, retching, and eventual insensibility, it claimed little notice in the train of symptoms.

"The complaint now clearly developed itself. The various affections to which she had been so long a sufferer were obviously attributable to some disease of the medullary column. The distressing headach, rushing of blood to the head, ringing in the ears, throbbing at the temples, and fits of insensibility; the sensation of acute pain, or of the pricking of pins and needles, shooting forward through the face and jaws, in the course of the branches of the fifth pair of nerves, or lower down in front of the larynx; the difficulty of swallowing; the shrill croupy breathing; the pain and cramp of the stomach or chest; the oppression, and the dry, loud, convulsive cough, were all readily referred to disease, or irritation of the cervical portion of the spinal cord. The extreme soreness and pain of chest and sides; the pain at the upper part of the sternum, shooting down the arms to the fingers, and producing distressing tingling; the occasional numbness of the arms; the symptoms of cardiac and pulmonic disease, appeared to depend upon some affection of the upper dorsal and lower cervical; and the abdominal pain, tenderness, spasms, pseudo-inflammatory attacks, and those of dysury, or total suppression of urine, or painful affections of the limbs, were at once traced to some altered state of the lumbar and lower dorsal portion. All the complicated, and it would appear whimsical, attacks of this strange malady seemed now simple and necessary results, and their alternations with one another merely indicated the shifting of the diseased action, to new points of the vertebral chain." (P. 10.)

The spinal tenderness now increased to such an extent, that, when the patient was moved, to have her bed made, "she was accustomed to throw all the extensor spinal muscles into action, and, by a violent effort, bring the whole spine into a state of rigid extension, to preclude the possibility of the slightest motion." (P. 12.) An issue was now inserted on each side of the second cervical vertebra, which relieved the pain of the forehead, face, and scalp. Fresh symptoms again appeared.

"Towards the close of February 1829, while drinking in the evening, she felt a sensation as if something gave way in her chest, as if the band from the upper part of the sternum, before spoken of, had snapt. She was instantly attacked with oppression, a sense of burning and pain in the throat and chest, croupy breathing, total loss of speech, and blindness of the left eye, with numbness and paralysis of the left arm; she had also a sense of numbness extending from the point in the chest where she felt the band snap, across to the shoulder, and down the left arm to the fingers, some difficulty of swallowing, and violent pain, straining, or retching, when the smallest quantity of food or drink reached the stomach. There was some swelling and excessive tenderness of stomach, with violent cramp at intervals, which extended down to the limbs and knees. The secretion of urine was suppressed, no more than half an ounce having passed in twenty-four hours, and that thick and black. There was no tenderness or fulness in the pubic region.

"After the lapse of some days, during which croton oil and diuretics had been freely used, the eye partly recovered its power, and the action of the kidneys was restored. Blisters to the throat and neck were of little advantage; but, on applying one to the occiput, some degree of voice was manifestly recovered, and the power of swallowing perfectly; the fingers of the paralysed arm also seemed to acquire a little motion. In July, a very decided improvement had taken place. The arm had attained much strength; and she was able to speak in a low whisper, though with pain and difficulty. It should be observed, that the power of articulating was never lost, so that, even while partly dumb, she could often make herself understood by a distinct, voiceless articulation of the words." (P. 13.)

A great amendment has taken place of late years: the patient is now cheerful, speaks perfectly well, and entertains hopes of recovery.

The introductory observations conclude with an abstract of what is to follow, which we shall present to our readers; for, as we intend to give them the essence of the whole banquet, it is but right that they should see the bill of fare.

"As, in the present state of our knowledge, strict distinctions, founded on the supposed nature of various spinal affections, must be liable to much error, it seems proper to offer such only as the symptoms would obviously indicate, without assuming that they are in all instances founded on any specific difference in the nature of the complaint. The following may be said to include all which have fallen within our experience.

"1st. Cases of irritation of the spinal cord, with tenderness at one or more points of the spine.

"2d. Cases with symptoms resembling the foregoing, but unattended by spinal tenderness.

"3d Cases of acute spinal inflammation, attended by pains of a rheumatic character, and by many of the symptoms of general irritation of the cord; but chiefly marked by high fever, excruciating pain and tenderness in some part of the back, occurring in paroxysms on the slightest motion, and often occasioning or ending in paralysis.

"4th. Cases of caries of the vertebral bones and distortion, which have been so ably treated of by many eminent writers, it is merely necessary to name, as much rarer diseases than any of the foregoing, but having very many symptoms in common with them, and affording frequent grounds for apprehension and error, when the diagnosis is not attentively studied.

"5th. The same may be said of those organic diseases of the spinal cord whose pathology Dr. Abercrombie has taken such pains to illustrate. We have met with very few of them in the course of our practice, and those were such as offered little that was new or interesting on the subject." (P. 25.)

The second chapter treats of *Irritation of the Cervical Portion of the Cord,* and the first section in it details numerous instances of affections of the sensitive system, proceeding from this cause. Thus, our authors tell us that

"Acute and chronic headach, browach, aching of the cheeks and face, pains in the breast or side, or sternum, or at the shoulder or down either arm, may be mentioned first, as among the most common symptoms of cervical irritation, both in the simple and complex cases. They are continually met with, as well as the subsequent ones of affections of the senses, in cases of organic disease of the cord, though then usually in connexion with others of a more formidable nature. The following are taken almost indifferently from our case-book.

"VIII. A young gentleman, aged twenty, complained of intense pain in the crown of the head and forehead, with excessive soreness of the scalp and feeling of general illness; is subject to attacks of the kind, and usually relieved by purgatives and lying down.

There was great tenderness of the five upper cervical vertebræ, pressure on any of them occasioning the pain in the vertex and brow. Purgatives and rest were again successful in relieving him; the application of leeches and a blister to the nape of the neck, to remove the tenderness, were then recommended. As long as this symptom remains, however effectual the relief, the complaint can only be considered as suspended.

"IX. James O'Brien, aged fourteen years, applied at the dispensary, complaining of pain and soreness in the crown and forehead, especially on stooping, sometimes very distressing, and attended with deafness. There was tenderness of all the cervical vertebra; pressure on the first or second excited pain in the vertex and brow. Was ill one year. Recovered by the use of purgatives, and of blisters to the nape of the neck.

"X. Ann Lynch, aged nineteen years, troubled with distressing headach, especially of the forehead, with sickness of stomach and thirst. Pulse ninety-five, tongue white, bowels confined; catamenia regular. Had been ill six days. Pressure on the first or second cervical, or behind the mastoid process, excited the pain severely at the brow. Was relieved by an emetic, followed by purgatives and a blister to the neck.

"XI. Mary O'Brien, aged forty years, ill three years, complains of pain in the head, particularly severe over the brows and at the temples, and occasionally confining her to bed for days. She is very weak and nervous, has no appetite, and is worse after eating. Is occasionally attacked with pain of stomach. On examination, there was found extreme tenderness of all the cervical vertebræ; pressure on any of them, or behind the mastoid process, exciting the pain severely at the brow and temples. There was also soreness of the seventh or eighth dorsal vertebra, pressure on which occasioned pain at the ensiform cartilage. In this case there was so much general debility, and so many points of the spine were affected for a length of time, that a rapid recovery was not to be anticipated. She did well after some weeks, by the strictest attention to the digestive organs, a course of tonics, and occasional small blisters to the spine.

"XII. Mrs. M., aged forty years, a nurse, complained of headach, soreness of stomach, and soreness and pain of chest, with stiffness at the right side of the neck. This stiffness increased suddenly at times, seizing the muscles like cramp, and followed by hoarseness and dimness of sight. Was debilitated, and in bad health. There was great tenderness on pressure at the middle cervical and seventh dorsal vertebræ. This patient was treated like the foregoing, and was also slow in recovering.

"The soreness of stomach was in all probability referrible to the tenderness at the seventh dorsal vertebra, and not to irritation at the trunk of the par vagum. It is then usually accompanied by sickness and loss of appetite.

"XIII. Catherine Deely, aged thirty years, six weeks ill, complained of constant distressing headach, with pain in the stomach and nausea after eating. Bowels are in a natural state, but sometimes griped; catamenia regular. Pressure on the dentata excited the pain in the forehead, and on the ninth dorsal, at the stomach. Recovered under the use of mild aperients, acids, and counter-irritation.

"XIV. Anne Day, aged thirty years, complained of headach, soreness of the whole scalp, frequent faceach, affecting the branches of the fifth pair of nerves; pain and tenderness down the neck and left arm, which rendered her unable to work; and pain between the shoulders at the left side of the spine. There was tenderness of all the cervical vertebræ, pressure on any of them occasioning the corresponding pains.

"XV. ———, a smith, aged thirty years, complained of pain at the outer part of the elbow between the external condyle and olecranon, which after a few days removed to the outer part of the arm, a little below the insertion of the deltoid muscle; there was neither heat nor swelling, but there was some tenderness in the affected spot; the pain was very distressing, often disabling him from working. There was acute tenderness at the two or three upper dorsal vertebræ." (P. 29.)

We know not whether it is from their property of soothing the irritation of the cervical part of the spine, but certain it is that blisters applied to the nape of the neck are among the best, if not the very best, remedies in obstinate headachs, which have set both purgatives and stimulants at defiance. Heberden mentions as the best remedies, a blister to the head, cupping near the occiput to the amount of six ounces once a month, and a pill consisting of one grain of aloes and four of calumba, taken at bedtime. (*De capitis dolore. Comment.* p. 87.)

A little further on we have a case of hemeralopia, proceeding from the same cause, and yielding to similar treatment.

"XIX. John Hayes, aged fifteen years, complains that, as soon as night falls, he invariably becomes blind: he cannot see the furniture or people about the room, when they are perfectly visible to every one else. The candle or fire-light appears a broad red haze, just distinguishable from darkness, but making nothing perceptible. He can perceive any dark object between him and the light, and no more. Has been affected in this way now about a fortnight, and had a similar complaint a year ago, which continued a good while. There is great tenderness evinced on pressing the second cervical vertebra. He perfectly recovered in less than forty-eight hours, by a small bleeding, an active calomel purgative, and a blister to the nape of the neck; and has since continued well." (P. 33.)

Several cases are then detailed of disordered vision; but, as their progress and event are not given, we shall pass them over, and quote one which is placed here, though it properly belongs to the chapter on general irritation.

"Margaret Doherty has been for many years suffering with intermittent ophthalmy and headach; the former always improving, or becoming worse, as the pain of head was lesser or greater. There was a granular state of the lids, with vascularity and muddiness of the cornea. She complained of pains in the neck and chest, and sometimes in all her limbs; general weakness, tremor, and numbness of the arms and legs. Her arms are often seized with sudden numbness and loss of power, so that she is in danger of dropping anything that happened to be in her hands. She was also subject to extreme coldness of the legs and thighs, and occasionally to faintings. There was general tenderness of the spinal column.

"All local treatment of the eyes was in this case wholly unavailing, but they gradually recovered by the use of general tonics and attention to the bowels. Whenever the head became worse than usual, nothing did so much good as large doses of the carbonate of iron. The eyes were always immediately benefited by it, and just in the proportion in which the pain of the head was diminished."* (P. 41.)

After some sensible remarks on the functions of the par vagum, which our authors suppose to possess common sensibility, several cases are given, in which vomiting appeared to depend on tenderness of the cervical vertebræ. In the first case, Michael Guerin, aged seventeen, was violently struck on the back part of the neck, about the fourth cervical vertebræ. He was seized with vomiting, attended by little or no nausea: this lasted four or five weeks, and then gradually abated.

Loss of appetite, as Messrs. Griffin justly observe, is so usual a symptom in all serious diseases, that it would be unsatisfactory to state cases of it, on the supposition that it depended on disturbance of the functions of the eighth pair; but this cannot be said, they alledge, of hunger or thirst, "which, occurring in the course of any disease, previous to convalescence, must be looked upon as symptomatic of some peculiar state of the cerebral substance at the origin of the par vagum." (P. 52.) Of course, as far as regards thirst, this passage must be understood with some qualification; for

"* To what are we to attribute the violent remittent ophthalmy described by Dr. Curry, some years since, in the third volume of the Medico-Chirurgical Transactions, as affecting himself? It resisted bleeding, blistering, purgatives, bark, the solution of arsenic, and all the usual plans of treatment; but got well at once by large and regularly-repeated doses of opium."

our authors cannot mean the thirst which ordinarily accompanies the pyrexiæ, but either one attended with hunger, or else a desire for stimulating rather than refrigerating liquids. Thus, in a remarkable case which they relate, of a young lady suffering under extensive spinal irritation,

"Some ale was brought to her, which she drank without stopping. She drank a whole bottle of Clonmel ale in a few minutes, besides wine, which she asked for repeatedly. She rested tolerably well that night. The next day, Thursday, she chose to get up and come down to the drawing room, where she lay on the sofa. She seemed weak, and complained much of her sides, particularly the left. She ate very heartily, however, and took two glasses of wine before dinner. At dinner she ate broiled mutton, drank a bottle of ale, and said that nothing but wine and ale could satisfy her." (P. 53.)

The remarks of Messrs. Griffin on this case are practical and useful.

"To understand this case fully, it should be mentioned that this young lady had, in her general state of health, a very slight appetite, and was never accustomed to more than the smallest quantity of wine or ale at any time. It was singular to see the usual order of things so completely reversed when she became ill; for, instead of losing her appetite, as others would have done, her hunger grew ravenous, and her thirst insatiable. This state we believe is connected with a feeling of nervous sinking, which is in some measure relieved by any thing, whether solid or liquid, taken into the stomach. It does not arise from direct debility, and would be best relieved by an opiate, followed by some aperient. It would not, however, be altogether safe to refuse giving some proportion of the stimulant, the little effect it has on the pulse or head being a tolerable proof that there is some need for it." (P. 54.)

The following is a good instance of this unnatural thirst, divorced from the fever which is its usual attendant.

"We shall never forget a case of sudden, unquenchable thirst, which we once witnessed in a child who was ill with irritation of spine; four or five of the lower dorsal vertebræ being swelled and tender, and occasioning the usual symptoms of tightness, pains in the bowels, and general delicacy. Having got some antimonial preparation to relieve a cough at the close of a severe illness, vomiting occurred, and she was soon after seized with spasmodic yawnings and craving thirst. No patient in the most burning fever ever seized the bowl with such wild eagerness of look, or drank with such unquenchable desire. Draught after draught was swallowed with rapidity, and still the eye glanced about with almost frantic impatience for more! more! At length she got some antispasmodic, ether and ammonia we believe, and became somewhat quieter, and in half an hour was perfectly relieved. During the attack the pulse was too quick to be counted. The child was not in the slightest degree feverish when it came on, which was quite suddenly, after the sickness of stomach and retching." (P. 55.)

Our authors at one time conjectured that a constant gastrodynia was a neuralgia of a dorsal nerve; while deep-seated spasmodic pain depended perhaps on a morbid state of the par vagum, and therefore was more frequently attended by nausea: but these suppositions have not been confirmed by their subsequent experience. They observe, that, "from the tables given at the close, one would be rather led to believe that the sickness of stomach depended on the cervical irritation,—the pain on the dorsal. In thirty cases of the former, there "was pain of stomach in only two. In forty-six of the latter, it was present in thirty-four." (P. 56, *note.*)

Our readers have probably begun to conjecture that we intend this to be a very long review, or rather analysis, of the instructive work before us; and they have guessed aright: but still, *est modus in rebus,* we must not reprint the whole book, and we shall therefore pass over the remainder of the section, merely mentioning that delirium, ocular spectra, and fits of insensibility, are also enumerated among the symptoms produced by irritation of the cervical part of the spine, and cured by appropriate treatment.

The second section is on *Affections of the Vascular System connected with Cervical Irritation;* and the following cases exemplify the important fact, how accurately functional disorder of the heart's action may simulate some of the most distressing symptoms of organic lesion.

"James Casey, a smith, had part of his ear bitten off in a quarrel. The inflammation and soreness were so great, that he could not sit up in bed; and, though a strong man, generally fainted when the sore was dressing. This did not excite much surprise at first, as it was attributed to the tenderness of the wound in a peculiarly sensitive habit; but when it began to heal, and all extraordinary soreness had worn away, it seemed very remarkable that he should still continue subject to sudden sinkings or lownesses, closely approaching to syncope. There appeared an extravagant disproportion between the apparent debility or nervous depression, and the trifling nature of the wound. When the lowness came on, he was always terrified by the apprehension of dying, and was obliged instantly to have recourse to wine and stimulants for relief; which, as he had no thirst nor heat of skin, were not forbidden. From the resemblance which these fits bore to the sinkings which are sometimes observed, in hysterical habits, in females after delivery, irritation of the cervical portion of the spinal marrow was suspected. On examination, there was found very great tenderness at the third and fourth cervical vertebræ, particularly acute at the right side. As the wound was now healed, and the disposition to fainting was much less frequent, it was thought unnecessary to institute any local treatment; attention to the bowels, and the volatile tincture of valerian, with camphor mixture, completed the cure.

"Mrs. ——, a lady, aged forty-eight, was awoke in the night by a sensation of weight and constriction across the chest; pain at the ensiform cartilage, and violent palpitation, followed by fits of sinking or fainting, with apprehension of dying. The palpitation was always brought on to a distressing degree when she chanced to turn on her left side. These symptoms continued to recur for some days, but were very much relieved by mild purgatives and antispasmodics; she was, however, now seized with acute pains in the neck, arms, chest, and sides: and, on examination, there was found tenderness of the first and second cervical vertebræ, and of the seventh or eighth dorsal. All these symptoms readily disappeared, on the application of a blister to the latter place; and her usual health was restored by a continuation of the antispasmodics, with tonics. The attack seems to have originated in fright and mental anxiety, and was readily reinduced, though in a slighter degree, by any new distress, for several months afterwards.

"Although we suppose, in these cases, that the primary disease exists in the cord, the ganglia are necessarily implicated. It is on them, and through them, the spinal irritation exerts its influence; and we may have the upper or lower ganglia affected, according as the irritation shifts its place in the spine. As an instance of this, we have been, within these few days, sent for by the lady whose case is just detailed, and found her labouring under a new train of symptoms. She had been seized, two or three times during the last week, with a sudden rush of blood to the head, which seemed to commence at the clavicles, and pass up in the course of the carotids. There was a momentary faintness, or tendency to insensibility, with loss of power of the arms; and she complained of occasional pain at the crown of the head and brow, sometimes occurring suddenly, and attended by stiffness and tenderness of the muscles at the back of the neck, especially at the right side; she had also slight cough, and an internal soreness of the chest, which she compared to the sensation experienced when a blister is taken off. She has had also a return of the palpitations at night. There can be no doubt of these symptoms yielding to the usual treatment." (P. 76.)

Our authors conclude this section by observing that, before the time of Corvisart, organic diseases of the heart were generally overlooked, and believed to be very uncommon; even obvious cases being treated as nervous disorders. Pathological anatomy produced a total revolution in medical opinion, and the opposite error was then committed of taking functional diseases for organic, and frightening dyspeptic patients with the supposition that their palpitations proceeded from structural and incurable disease. This error is again disappearing, and we must therefore be cautious how we fall into the old one again, and overlook organic disease, as our medical ancestors too often did. The symptomatic diseases of the heart are infinitely the most numerous, but the structural ones are by no means rare.

The third section treats of *Affections of the Respiratory System connected with Cervical Irritation.* The most common symptom belonging to this subdivision is a dry cough. The first case is one of a young lady, aged seventeen, suffering from a short slight cough, with a pulse of 120, a hectic tint on the cheek, and tenderness at the lower part of the sternum. "There was tenderness of all the cervical, and of the four upper dorsal vertebræ; pressure on any one of which instantly brought on coughing." (P. 87.) Ten leeches and long narrow blisters, applied to the tender vertebræ, formed the most efficacious part of the treatment, and the patient was cured in two or three weeks, the cough abating as the tenderness disappeared.

In the next case, a young lady, of the same age, became affected with great pain in the right side, great tenderness on pressure, sickness, and feverishness. "On examination of the spine, great tenderness of the second cervical was discovered: pressure there occasioning acute pain in the vertex and brow; pressure on the lower cervical and upper dorsal excited pain there, and loud coughing; at the seventh or eighth, the same symptom, with pain of chest; and at the four last dorsal, as well as at the margin of the ribs, as far forward as the ensiform cartilage, there was extreme pain on pressure." (P. 88.)

A variety of treatment was resorted to with temporary benefit, but there were two relapses. Of the last set of remedies employed, the most beneficial consisted of stimulating liniments applied to the spine. The patient declared that they did her more good than all that had been made use of from the beginning. Our authors proceed to say,

"The hard, barking cough, which Dr. Clarke describes as affecting young females, and which yields to sudden effusion of cold water, after foiling every other remedy, was, we should suppose, a mere symptom of spinal irritation. Tenderness of the vertebræ would have been discovered had any examination been made; nor, indeed, can we imagine a disorder of any other nature likely to be so suddenly and so perfectly relieved. The cough remaining after the acute stage of hooping-cough is over, which is said to depend upon habit, is also perhaps dependent on an irritable state of the cord.* Since the conjecture occurred to us, however, we have met with no instance to confirm it. In typhoid, inflammatory, and still more frequently, in intermittent fevers, cough is often a symptom of nervous disorder, and especially disorder of the upper part of the spinal cord. It is the more necessary for the practitioner to have this continually impressed on his mind, as, from its connexion in these cases with high febrile excitement, it may very readily lead him to imagine he has to contend with local inflammation." (P. 89.)

The symptom next in frequency to cough is oppression of the breathing, varying through a thousand grades, from a slight dyspnœa to the most terrific asthmatic paroxysm. It is to cases of nervous asthma, and perhaps to these alone, that galvanism, as suggested by Dr. Wilson Philip, is applicable.

The following remarkable case shows the relief obtained by applying the remedy to the origin of the disease.

"A young lady of an asthmatic constitution, and whose habit was so susceptible that town air or a close room instantly occasioned dyspnœa with piping respiration, caught a severe cold, and was in consequence attacked with a violent paroxysm, attended with considerable fever. She was found with purple cheeks and lips, supported in the bed by pillows; the chest heaving; the muscles of respiration tense and labouring; the pulse was 120, small and compressible. On examination, slight tenderness was discovered at the two or three upper cervical vertebræ. Together with other remedies usual in asthmatic cases, a blister was applied to the neck, much to the surprise of the patient, who had been always before blistered on the chest. Perfect relief to all the symptoms, but especially the oppression, was obtained; and the paroxysm on the next night was scarcely observable.

"In a few weeks afterwards, she had a return of the fit in a more violent degree, and applied two large blisters in succession to the chest, at her own counsel, without the slightest benefit. Her medical attendant was now sent for, and found her, if possible, in a much worse state than on the former occasion. A blister was again applied to the neck, and a mild diaphoretic mixture, with hyosciamus, ordered. Though not effecting so complete a resolution of the paroxysm as in the former instance, it produced a surprising mitigation of the disorder. The young lady and her friends were particularly struck with the obvious relief which the remedy procured.

* "The tussis spasmodica, which Underwood describes as [affecting infants, remaining dry and hoarse under the use of pectoral remedies, but soon relieved by opiates or cicuta, is evidently of this nature. Perhaps the same may be inferred of the cough described by Dr. Gregory, of London, as dependent on an irritable state of the mucous membrane, which he describes as not benefited by any remedies which he has been able to devise, except change of air."

" It must be evident that leeching, blistering, or friction with liniments at the origin of the nerves, can only be of use in the special instance of nervous disturbance; and perhaps we might say, generally, only where spinal tenderness is to be met with." (P. 91.)

A great part of this section is occupied with a refutation of Dr. Cheyne's opinions on croup. He believed that there was but one kind, for, from the supposed identity of the symptoms in the spurious and the inflammatory croup, he conceived them to be but varieties of the same disease, and advised that we should act as if the spasmodic kind did not exist. But the truth is, that in the nervous croup there is an intermission in the symptoms, which is of itself sufficient to establish a most important distinction. We must refer our readers to the work itself for Dr. Griffin's judicious answers to the questions on croup addressed by Dr. Cheyne to Dr. Kellie; and shall pass on to the following interesting and well-written remarks.

" Dr. J. Clarke believes that this, and indeed all other convulsive affections of children, depend upon some organic affection of the brain. He details the post-mortem appearances of a few cases in illustration, and says that ' all the arguments founded on the doctrine of sympathy and irritability are drawn *ab ignoto;* and it seems much more conformable to reason and observation to infer that such convulsive affections arise from some derangement of organization, however temporary, than to resort for an explanation of them to imaginary causes, and such as offer to the mind no satisfactory conclusions.'

" In reply to this reasoning, it may be remarked, that our knowledge both of the physiology of the brain and spinal marrow, and the pathology of its many diseases, is far too obscure to allow of our drawing any inferences not warranted by established facts. It is surely more philosophical to infer change of structure only where we find it, and to suppose some other state capable of disordering the functions of parts, where we do not find it, especially when such conclusions seem strikingly confirmed by a fact that might almost suggest itself,—the slowness, the imperfection, or impossibility of cure in the former; the suddenness, and perfection, and facility, with which it is often accomplished in the latter. No one is so ridiculous as to suppose that no change takes place in functional disorder; but it would certainly seem that, in such as are said to depend on irritation, no change of structure takes place, no deranged organization. A person ascending in a balloon, at a certain height becomes oppressed in consequence of the rarity of the air, not from any change or breach in the mechanism of his frame, but because of the altered relation between that frame and the atmosphere.

" We have felt it necessary to dwell a little on this subject, from a conviction of the great importance, in all disorders of the system, of distinguishing the organic from the functional. We are quite of Dr. Underwood's opinion, that, even when convulsive affections prove suddenly fatal, they are most commonly sympathetic, or dependent on irritation; and in believing this, we are treasuring up for ourselves new hopes for our remedies, and increased zeal in their application." (P. 103.)

This section concludes with some observations on hydrophobia, which our authors believe to be a disease whose seat is in the upper part of the spinal marrow, and particularly that portion of it which is allotted to the function of respiration.

The fourth and last section of this chapter treats of *Affections of the Motor System connected with Cervical Irritation.*

The most interesting portion of it consists of several cases of epilepsy cured by treatment directed to the spine. The result of the first case was so encouraging, that Dr. Griffin sent a message to a young girl who had been attending at the dispensary, with the same disorder, but had been dismissed uncured two years before. On her return, she stated that she had now had three fits a week for four years and a half. In this, as in the former instance, there was cervical and dorsal tenderness. "A pint of blood was taken from the temporal artery, calomel and saline purgatives were administered, and a blister was applied to the nape of the neck. She was instantly relieved, and from that time to the present (several years) has not had a single fit. Whenever any threatening symptoms occurred, similar treatment was immediately pursued." (P. 111.)

Another case is then given, in which similar treatment was successful; but Dr. Griffin adds, that he has since met with epileptic patients, chiefly men, in whom no tenderness of the spine could be discovered, and with whom every variety of treatment was unsuccessful.

The third chapter treats of *Affections produced by Irritation of the Dorsal Portion of the Cord.* This chapter is a short one, as, much that might have found a place in it has been anticipated, the dorsal vertebræ having been often implicated in the cervical cases. The following are some of the more interesting cases under this head.

"A lady of delicate constitution had been for a considerable time, as she was informed by her medical attendant, labouring under an affection of liver. She had constant troublesome pain and soreness in the right side, beneath the short ribs, and sometimes up between the shoulders; there was slight disorder of the digestive organs. She applied to us, for the purpose of being put under the influence of mercury; but, on examining the spine, we found considerable tenderness about the ninth or tenth dorsal vertebra, which there was reason to suppose occasioned the affection of side, as the patient's countenance indicated no very serious disease. She was therefore ordered mild purgatives, and a blister to the tender vertebra. The complaint, which had annoyed her for months, was thus, to her great astonishment, perfectly cured in a few days.

"It is always useful, in these doubtful cases, to examine the opposite side, in which it will be often found symptoms precisely similar exist,—a tolerable assurance that they are, in neither one nor the other, connected with the state of the liver." (P. 124.)

"Catherine Williams, aged twenty-five, was seized with sudden violent pain at the pit of the stomach, succeeded by a feeling of lowness, and at last by stupor, loss of speech and motion. She became better towards night, but had a return of the attack in the morning. The lowness and stupor eventually came on in fits, preceded, not by the pain of stomach, but by general shiverings. In these fits she lay, with her eyes closed, moaning, and sensible to every thing about her when roused or excited, but incapable of speaking. The pupils of the eyes were not dilated. Whenever she took drink, she was attacked with hiccup and flatulent eructations to a distressing degree. Her skin was cool; tongue clean; pulse ninety, but variable; catamenia regular. There was no tenderness of the cervical vertebræ; but she complained, when pressure was made about the eighth or ninth dorsal. After free purgation, she slept well; but the fits recurred on the succeeding morning, with cramps or contraction of the fingers of the right hand. As the purgatives gave no permanent relief, a blister was ordered for the tender part of the back. She neglected to have it applied, however, and passed another day in the same state as the preceding. She then submitted to the blister, and had a rapid amendment. The aperients were continued throughout the treatment.

"We remember to have seen a case precisely like this, occasioned by general nervous irritation, in advanced pregnancy. The fits of insensibility were put an end to, by the fright at an attempt to bleed her, which, however, she did not permit; and the other symptoms were relieved by aperients and antispasmodics.

"We shall only offer one case more, in illustration of these dorsal affections; which, if it is to be looked on simply as an instance of irritation, should impress us with the necessity of examining the vertebral column in all chronic as well as acute abdominal pains.

"Michael O'Donnel, aged forty-five years, had been affected for three years with pain at the right side, about the situation of the colon, but confined to a very small spot. It intermitted very little

T 2

during this period; and occasioned much flatulence, loss of appetite, and emaciation. On examining the spine, pressure on the ninth dorsal vertebra brought on the pain in the colon, and eructations; the pulse was natural, and the tongue whitish. We find no record of the progress of the case." (P. 126.)

The fourth chapter treats of *Affections produced by Irritation of the Lumbar Portion of the Cord.*

It has often been justly remarked, that the progress of knowledge, while it increases the number of things to be learned, and thus makes their acquisition in one respect more difficult, on the other hand, by simplifying and classing, and showing that facts long thought to be distant from one another as pole from pole, have in reality a secret link between them, renders recollection as well as analysis more easy. And thus many of the neuralgiæ, the hysterical knee, the irritable uterus, the pseudo-hepatitis, the croup which requires neither leeches nor calomel, not only arise from one common source, but may be detected in the same manner. These reflections naturally pass through the mind of him who peruses the following passage, where the illustrious names of Abernethy, Brodie, and Gooch are appended to discoveries which formerly were isolated, but are now connected by a link alike true in theory and serviceable in practice.

"If functional affections become more interesting as they bear a closer resemblance to organic diseases, there are very many belonging to the lumbar portion of the cord which claim particular attention. They were in fact continually confounded in general practice, before the late discoveries in physiology, although a few eminent men began at an earlier period to detect and distinguish them. Mr. Abernethy, very many years since, pointed out the existence of a disease, simulating an affection of the vertebral bones, and yet not of that nature, but, as he believed, a nervous disorder dependent upon disturbance of the digestive organs. Mr. Brodie, as has been already mentioned, published cases resembling caries of the hip-joint or ulceration of the cartilages, in which no such complaint existed. He considered them as hysterical affections. Dr. Gooch gave interesting accounts of painful complaints of the uterus, unconnected with structural or inflammatory disease of that viscus. These, with numerous other disorders, are now so well known as to form a class of neuralgic affections; but they are still for the most part looked upon either as idiopathic, or as symptomatic of irritation in some distant organ, and are seldom attributed to the source we are endeavouring to trace their connexion to,—disturbance of the spinal marrow.

"We may consider these disorders, like those of the cervical or dorsal portion of the cord, as consisting in preternaturally increased sensibility or action, or in a diminution or loss of either. Among the former, we have pains in the sides or abdominal parietes, colic, pain in the kidney or bladder, or uterus or ovarium, or in the spermatic cord or testes, pains in the joints or muscles resembling rheumatism, or ulceration of the cartilages of the knee or hip-joint. Again, we have cramps in the bowels or legs, or feet, or we have diarrhœa, leucorrhœa, or menorrhagia. Among the complaints marked by loss of sensibility or power, is a sense of weight or fulness of the abdomen, with flatulence, and perhaps obvious distention. This would seem to depend on loss of sensibility in the intestines themselves, and is generally connected with obstinate costiveness. It is the state sometimes induced by the administration of the carbonate of lead. There is another of the same nature in which the spinal nerves are those chiefly affected, denoted by diminished sensibility of the abdominal muscles. Such is the case with persons who feel as if their bowels were falling out, or with those who feel like the gentleman described by Mr. Abernethy, as if they had no bowels. We have also in the same class defective or suppressed secretions, and partial or total paralysis of particular muscles or organs." (P. 166.)

The following cases are important as well as curious, and the reflections to which they give rise are stamped with the practical good sense which distinguishes the work before us.

"M. H., aged eighteen, ill one year with pain over the crista of the left ileum, extending forward to the umbilicus; it was only occasionally distressing to her, and was always relieved by lying down. The menses have been suppressed since she first complained. There was tenderness of the middle lumbar vertebræ.

"Mrs. L., aged fifty years, complained of extreme pain and soreness over the whole abdomen; exquisitely acute at the right side of the umbilicus. The pain came on in violent paroxysms; there was no thirst or feverishness; pulse eighty, and feeble. On examining the spine, we found acute tenderness of all the lumbar vertebræ, especially the lower ones. On pressing them even lightly with the finger, she screamed aloud, and implored us not to touch that part again, as she could not bear it. She speedily recovered by the employment of purgatives and fomentations, and the application of a blister to the spine.

"Instances of these abdominal pains, dependent upon spinal disorder, might be multiplied without end. It is indeed scarcely probable that they could be absent in any case in which the lumbar portion of the cord, or its membranes, are severely affected. This is exceedingly well illustrated in rheumatic complaints of the spine, where neither the nature nor the seat of the complaint can be matter of any doubt. A gentleman stooping in dressing himself, to draw on his stocking, was seized with pain about the ninth dorsal vertebra, as if his back was broken, or the spinal column was dislocated at that point; he had some difficulty in getting to bed, and could not draw a full breath, on account of violent pain at the insertions of the diaphragm; the pain afterwards extended down the spine, affecting the lowest dorsal or upper lumbar vertebræ, but the chief suffering was from pain at the right side, close to the umbilicus, and a little lower. This continued in a very acute degree, even after the pain of back and difficulty of inspiration yielded to bloodletting, anodyne liniments, and the volatile tincture of guaiacum. Though the patient was a medical man, and was aware the abdominal pain was superficial, arising from the morbid action at the origin of one of the spinal nerves, he had much ado at times to convince himself it was not deep-seated and in the bowels, like colic. In fact he could not do so, if it was not for the undisturbed state of the digestive functions, and that the colic or pain was instantly brought on in a violent degree by the slightest unwary turn or twist of the spine. It was the last symptom of the rheumatic attack which yielded; and this naturally led him to consider, if the complaint had set in precisely in the form which it assumed towards its termination, how puzzled, and probably deceived, he would have been.

" Dr. Pemberton, many years since, in treating of inflammatory affections of the kidneys, spoke of the sympathetic soreness in the abdomen, with which they were often attended, as likely to lead to a misconception of the seat of disease; but it is obvious from what has been said, that both abdominal soreness and pain in the situation of the kidney might exist, without the presence of any serious disease either there or in the abdomen, if the cord be in a state of disorder; and we venture to assert, no practitioner can, in any doubtful or obscure case, assure himself from the danger of mistake, who neglects an examination of the vertebral column.

" Though these sympathetic pains are usually superficial, and seated in the abdominal parietes, it seems probable that true colic (a true spasmodic affection of the intestines, through the medium of the ganglia of the sympathetic,) might be occasioned by irritation of the spinal cord. This has been the opinion of many of the continental writers with respect to Colica pictonum, but strong analogies may be drawn from the affections incident to spinal irritation, which would lead us to conclude that other morbid states of the spinal marrow, beside that apparently induced by lead, may excite spasmodic stricture of the bowel. We recollect to have met with a very violent case of colic following an injury of the lower dorsal or upper lumbar vertebræ, by a fall. It was relieved by a large bleeding and by blistering the spine. As the man had, however, been affected with colic at former times, when no such injury had been received, a satisfactory inference could not be drawn from the facts stated.

" Accompanying these painful affections of the abdomen, especially when they assume the form of griping, we frequently have diarrhœa; as in preternatural excitement of the kidneys we have a superabundant flow of water from the bladder. That diarrhœa may be brought on by an excited state of the cord, we have ample proof, in its occurrence among a successive medley of complaints, in cases where there was no disease anywhere but in the spine,—those of general irritation. We recollect a remarkable instance of protracted spinal disorder, in which pain and tenderness in the lumbar vertebræ alternated with a similar state of some of the lower dorsal or upper cervical. There was a corresponding change in the patient's complaints, from menorrhagia or diarrhœa, with tenesmus, to sickness and pain of stomach, and eventually to distressing toothach. The following is the only uncomplicated case among our notes, and seems well marked.

"Margaret O'Donnel, aged thirty years, was attacked suddenly, last week, with diarrhœa and frequent pains in the bowels. She had been previously in good health. There was tenderness of the first or second lumbar vertebræ. *Pressure on them brings on the pain in the bowels which usually attends the fit of diarrhœa.*" (P. 130.)

The other affections depending on lumbar irritation detailed in this chapter are, cholera, symptoms of gravel, irritable bladder, irritable testis, constipation, suppression of urine, and paralysis of the lower extremities.

In a very remarkable case of suppression of urine, arising from a fall on the spine, two or three drachms were drawn off by the catheter on the seventh day after the accident, and six ounces on the tenth; the urine had not been otherwise evacuated, yet the patient recovered. (P. 142.)

In the fifth chapter, *General Irritation of the Spinal Cord* is discussed.

Perhaps the most interesting cases in the work are those in which pressure of a particular spot in the spinal column brought on a fit of insensibility, or a thrill more unbearable than pain. As, for instance,

"Anne Hannan, aged twenty-two years, complained of pains in all her limbs and joints, pain at the sternum and sides, pain round the hips, and in the whole length of the back, from the neck down. Her complaints were relieved by lying down, but were increased excessively at night, although lying in the horizontal posture. The catamenia, which had been suppressed for twelve months, returned since her illness. Her appetite was bad, and she complained much of debility. There was great tenderness of all the dorsal and lumbar vertebræ, but none of the cervical. Pressure at the upper dorsal occasioned pain at the middle of the sternum; from the third or fourth down to the sacrum it excited pain, not at the corresponding points as usual, but at the ensiform cartilage. Pain was even brought on in this situation by pressure behind the trochanter, or on the muscles of the thigh, or on the knee-joint, or if she chanced to tread on uneven ground, or a pebble came beneath her foot in walking.

"To ascertain whether the seventh or eighth dorsal vertebra was as usual more affected than other parts of the spine, we were induced to make rather firm pressure there; when she suddenly tumbled forward in a fit of insensibility, and would have struck her face against the floor, had she not been caught by a person who stood near her.

"A lady, aged thirty years, complained of pain affecting the left side of the face, temple, and neck, which she supposed to be something of tic douloureux, and which had annoyed her for two or three years. She had been occasionally subject, during the same period, to pain of side, to leucorrhœa, dysmenorrhœa, dysury, with bearing-down pains and incontinence of water. On examining the spine, which she had no conception was at all affected, considerable tenderness was found about the seventh dorsal vertebra, and at all the lumbar; but, on touching the second upper vertebral bone, which, from the symptoms affecting the face and neck, there was reason to suppose was also tender, she sprung up with frightful suddenness, as if a needle had been driven through the cord, and then as instantaneously fell back in a state approaching to insensibility. Out of this stupor she sprang twice with the same electric suddenness, and as often fell back powerless, her countenance during the moment evincing the utmost terror and agitation, and her respiration becoming heaving and oppressed. As soon as she could speak, (which was at first in a broken, affrighted manner,) she said that, the instant her neck was pressed, her arms, and all parts of her person above the ensiform cartilage, felt as if suddenly numbed or paralyzed. There was a numbness and sensation as from the pricking of pins and needles, tingling through her face, jaws, temples, and arms, to the tips of her fingers. She had never experienced such a sensation before, and would on no account permit her neck to be touched again. As a proof of the functional nature of this lady's complaint, we may mention that, although she adopted no regular plan of treatment, and did little for herself, her health did not become worse, and she afterwards married and had a family.

"A young gentleman described himself as suffering for some years with chronic liver complaint. He had constant but variable pain in the right side, with general delicacy of health, and had taken blue pill and other medicines without relief. He had, however, derived considerable benefit from drinking the Liston Varna waters, which are strongly chalybeate. Some days since, he was seized with vertigo and loss of sight for a few minutes, accompanied by a thrilling sensation down the arms, and followed by a slight attack of feverishness. It was relieved by purgatives.

"Having strong doubts as to whether his liver was diseased, the right side was particularly examined. He complained of pain there, but there was neither hardness nor soreness. On examining the spinal column, although there did not appear to be any tenderness, the sensation of pressure was excessively disagreeable to him through its whole course. When the finger rested on one of the dorsal vertebræ, he grew pale and terrified, and would have fainted, if the pressure had been continued. He felt no pain, but a sudden indescribable sensation or thrill through every nerve in his frame, which was inconceivably horrid. He shuddered at the idea of permitting a repetition of the pressure, and had an unpleasant feeling about the part for the remainder of the day. When a few weeks had elapsed, however, he allowed another examination, and with precisely the same results." (P. 148.)

In one very curious case, where there was tenderness of the whole spine, the sensibility of the skin was universally diminished, and the patient complained that "he felt as if he had a cover all over his body." He also had a sense of weight along the spine, with numbness of the feet and hands; and "suffered from pyrosis, gastrodynia, headach, oppression [of the breathing], and weakness."

The chapter concludes with several "instances of metastasis," or cases in which the disordered action was transferred from one part to another, as the spinal tenderness shifted its situation. One lady, aged forty, who suffered from an infinite and most perplexing variety of uneasy sensations, was at one time tormented with an insatiable desire of having everything done with prodigious celerity. Dr. Darwin, had he lived in these days, would have put down in his Mat. Med. for such cases "travelling on a railroad."

The sixth chapter, on *Spinal Irritation resembling Inflammatory or Febrile Attacks*, is very short, but very important. Dr. Griffin says,

"A case which occurred in the clinical ward of the Edinburgh Infirmary, during our attendance there, gave a particular interest to our inquiries into the nature of complaints which simulate inflammation. A young woman was brought into the ward with, it was supposed, inflammation of bowels. She was bled very largely, and had, I believe, large doses of opium, with but little alleviation of the symptoms. On the second or third day, however, globus hystericus and other nervous symptoms supervened, which induced the attending physician to adopt an immediate change of treatment. In his subsequent clinical lecture, he stated, that hysteria sometimes so perfectly imitated an attack of inflammation, that it was absolutely impossible to distinguish between them. He recommended it therefore as the safest rule, in all doubtful cases, to consider and treat them as inflammatory, until some symptoms occurred which clearly marked them as hysterical. Of the prudence of this recommendation, under such circumstances of admitted difficulty, there could be no question; but the difficulty itself is, after all, not the less discreditable to our pretensions in diagnosis, and the alternative is certainly not always free from danger. It unfortunately happens that temporary relief generally follows even the free use of the lancet in hysterical or nervous affections, although the complaint is eventually rendered more obstinate, and the disposition to relapse is increased by it. But, in the broken, delicate habits in which such disorders frequently occur, it must be obvious the evil consequences cannot always be limited to a mere aggravation of the malady. Indeed, all physicians of experience will readily acknowledge that large depletion, in these cases at least, must frequently lead to irreparable mischief. The lady (Case ii.) nearly lost her life by the repeated abstraction of blood and by purgatives; and there is a case precisely similar, related by M. Jolly, in his Essay on Visceral Neuralgiæ.' The patient had a succession of attacks, resembling gastritis, hepatitis, nephritis, hysteritis, &c., each of which gave way for two or three days to the usual depletory measures; but the intervals of relief were short and imperfect. When at length reduced to the most extreme state of weakness and emaciation, the sulphate of quinine was administered in large doses, and the recurrence of the paroxysm prevented." (P. 160.)

We recollect a case which was sent into St. George's Hospital, during our attendance there, by a distinguished physician, on the supposition that it was one of acute hepatitis, but it turned out to be merely hysteria, in a girl of a sanguine temperament. Dr. Griffin acknowledges the possibility of transmission of irritation from an inflamed part, but thinks it rare: his remarks however on this and a kindred difficulty are so good, that we will not deprive our readers of the benefit of seeing them in his own words.

"The transmission of irritation from parts in a state of high inflammation is however, we believe, rare; while its transmission from a part in a state of irritation is of daily occurrence. Hence it is, that, in inflammation of the jaw from accidental inflammation, we rarely have constitutional symptoms; but in dentition, which is a state of irritation and not inflammation, we have vomiting, purging, cough, croup, convulsion, &c. Hence, in gonorrhœa, all the symptoms are confined to the neighbourhood of the inflamed membrane; but in the introduction of a bougie, which occasions irritation only, we have perhaps rigors or syncope. The same is true of the intestines. Violent inflammation occasions symptoms directly related to the parts inflamed; but the irritation of a worm excites frightful convulsions. Were we to attempt any explanation of these extraordinary facts, we might say, that the intensity of action and sensation in an inflamed part engages the nervous influence too powerfully to admit of distant minor effects; while the peculiar and less engrossing disturbance, the mode of sensation, if it be such, which constitutes irritation, is merely sufficient to act as an excitant to the central nervous masses. They seem, in short, to depend on the same law in the system, to which Dr. Whytt and Professor Alison have referred sympathetic actions, and on the admission of which we can understand why irritating the fauces with a feather, or the mucous membrane of the nose with mustard, should excite nausea, retching, or sneezing, when painful inflammation of these parts cannot produce such effects.

"There may perhaps be another source of fallacy in forming a diagnosis, besides the occurrence of spinal tenderness as a consequence of inflammation in peculiar habits,—the possibility of its existence previous to the inflammation. This may merit consideration, but is not of very great importance: since, as we have heretofore mentioned, pure inflammation is not at all common in those nervous or hysterical habits in which spinal disorders so readily occur." (P. 162.)

Of the pseudo-inflammatory attacks detailed in this chapter, one was hepatic, three uterine, and one enteritic. They are instructive, but so much has been written on this subject of late years, that we shall abstain from quoting any of them.

The seventh chapter exhibits these important affections in a totally different point of view; for it treats of *Cases resembling those of Spinal Irritation, but unattended by Spinal Tenderness, and perhaps referable to Irritation of the Ganglia of the Sympathetic Nerve.* Our authors here give a few cases, being all that they have met with, among so many of the neuralgiæ, unaccompanied by tenderness of the spine. In the first case, a woman, aged thirty, after the subsidence of an attack of cough, sore-throat, and feverishness, was seized, first, with a general œdema, loss of appetite, thirst, nausea, and a burning pain in the stomach and abdomen, without tenderness on pressure; afterwards, with ischuria, dysphagia, and globus hystericus. Purgatives afforded most relief, and she was cured in four weeks.

In another case, Dr. Griffin was called to see a young woman, who was supposed to be dying, but who was in reality in an hysterical fit. She likewise had some difficulty in swallowing, but no tenderness of the vertebral column.

The following instance is given as one of an obscure affection of the cord.

"Mrs. ———, was seized with violent pain about the sacrum, hips, and thighs, and subsequently in the calves of the legs and toes. It was of so distressing a nature that she could not rest a moment with it, but was compelled to be up and walking the house the whole night. The pain was not present at the same moment in all these parts. At one time it continued in the hips and thighs for some hours, at another in the legs or toes; the parts which were freed from the pain for the time feeling weary and sore. It came on generally about three o'clock in the day, continued severely for the night, and abated towards morning. She made use of the carbonate of iron, sulphate of quinine, henbane, purgatives, liniments, and fomentations, without advantage. The warm bath gave some relief, and opium usually procured some sleep; but neither remedy prevented the recurrence of the pain on the succeeding day. A large blister was at length applied to the sacrum, and on the next day there was no return of the pain; but, singular to say, at the usual hour of its attack, her legs were affected with an uncontrollable restlessness, and she was forced to keep up a continual sort of kicking motion with them as she sat in her chair. This, however, eventually subsided. There was not the least tenderness discovered either in the spinal chain or sacrum throughout the attack." (P. 178.)

This is obviously one of the most doubtful cases, and there seems to be but little reason for referring the symptoms to the cord; for as the spinal marrow, exclusively of its relations with the brain, has also a sort of independent existence of its own, so it seems not improbable that the nerves may have a like privilege, and that this was a genuine case of idiopathic neuralgia.

In speaking of *Cases of Acute Spinal Inflammation,* the subject of the eighth chapter, Dr. Griffin supposes them to have a "rheumatic origin." He does not mean by this that they occur in rheumatic subjects, but that they are cured (at least the cases which have occurred to him,) by less depletion than a pure inflammation of such an important organ would require. Perhaps a comparison to rheumatism may be objected to, as the pathology of that disease is quite unknown, and it is therefore explaining *obscurum per obscurius.* But Dr. Griffin's practical directions are equally valuable, whether we call this myelitis an erysipelatous or a rheumatic inflammation: he has found, he tells us, not only that these cases require less bleeding than ordinary inflammation does, but that they are benefited by the use of colchicum.

We now come to *a Tabular View of* 148 *Cases of Spinal Irritation, including all its forms.* This extends over sixteen pages, and forms a valuable abstract of the whole book. Appended to it is a summary, which contains in a condensed form so many important facts, and will supply every diligent practitioner with so many materials for thinking, and so much stimulus to observation, that we should be inexcusable if we did not quote it.

| "Cases. | Prominent Symptoms. |
|---|---|
| A. 28 Cases of cervical tenderness. | Headach, nausea or vomiting, faceach, fits of insensibility, cough, affections of the upper extremities. |
| 8 Men. | In 2 cases only, pain of stomach. |
| 8 Married women. | In 5, nausea or vomiting. |
| 12 Unmarried. | |
| B. 46 Cases of cervical and dorsal tenderness. | In addition to the foregoing symptoms, pain of stomach and sides, pyrosis, palpitation, oppression. |
| 7 Men. | In 34 cases, pain of stomach. |
| 15 Married women. | In 10, nausea or vomiting. |
| 24 Unmarried. | |
| C. 23 Cases of dorsal tenderness. | Pain in the stomach and side, cough, oppression, fits of syncope, hiccup, eructations. |
| 4 Men. | In 1 case only, nausea or vomiting. |
| 6 Married women. | In almost all, pain of stomach. |
| 13 Unmarried. | |
| D. 15 Cases of dorsal and lumbar. | Pains in the abdomen, loins, hips, lower extremities, dysury, ischury, in addition to the symptoms attendant on tenderness of the dorsal. |
| 1 Man. | In one case only, nausea. |
| 11 Married women. | |
| 3 Unmarried. | |
| E. 13 Cases of lumbar tenderness. | Pains in the lower part of the abdomen, dysury, ischury, pains in the testes or lower extremities, or disposition to paralysis. |
| | In one case only, spasms of stomach and retching. |
| F. 23 Cases, all the spine. | Combines the symptoms of all the foregoing cases. |
| 4 Men. | |
| 4 Married women. | |
| 15 Unmarried. | |
| G. 5 Cases, no tenderness of spine. | Cases resembling the foregoing. |

" In all, making 148 cases; 26 of which were males, 49 married women, and 73 girls."

These tables are excellent examples of what M. Louis calls the numerical method, on the importance of which he justly insists, as the chief or only method by which medicine can ever hope to aspire to the rank of a science. When facts are thus accumulated, and placed in juxta-position, it only remains for the enlightened physician to draw his inferences, and this Dr. Griffin has done with his usual ability.

An ingenious author observes, that the great Montaigne, in the abundance of his riches, frequently throws away in a parenthesis some remark, of which a more meagre writer would have made an essay, or perhaps a book; and thus Dr. Griffin, among the *Concluding Observations* which form the subject of his ninth chapter, has given in a note a case which many a distressed bookmaker would have expanded into a treatise on the therapeutic effects of fright. The remarks by which it is introduced are so candid and philosophical, that we shall cite more than the bare case.

" When typhus fever was very prevalent in this country, some years ago, we had opportunities of trying all the popular remedies for cutting it short in its earliest as well as its confirmed stage,—emetics, diaphoretics, purgatives, cold bathing, &c., but we could never convince ourselves that any particular plan of treatment was capable of arresting the complaint. Under all of these remedies patients occasionally got immediately well; but a vast majority of cases wholly resisted their influence. As we could attribute this failure to no observable difference, either in the treatment or the period of time at which it was employed, and as it seemed somewhat unsatisfactory to assume that remedies, which were inefficacious in ninety-seven cases out of a hundred, were yet successful in the remaining three, we necessarily concluded that those three were not instances of contagious fever at all.

"The only circumstance which would, notwithstanding these facts, induce us to believe fever might be interrupted or arrested in its course, like ague, by the influence of medicine, is our having witnessed the actual accomplishment of such a cure by a fit of terror.

"A girl of the name of Dalton, was visited, in the neighbourhood of Pallas-Kenry, as a dispensary patient, and being found in bad typhus fever, was transmitted to the Limerick Fever Hospital. In a week afterwards her brother took ill in the same house, and, after some days' illness, was visited from the dispensary. He was found confined to his bed with all the symptoms of confirmed typhus, and was also sent to Limerick. On getting out of the car, at the gate of the hospital, he was assisted up stairs by the nurses, but in his way was met by some persons who were descending with a coffin on their shoulders. The sick man inquired whose body they were removing, when one of the bearers inadvertently answered: 'A girl of the Daltons.' The brother, horror-struck, sprung from between his conductors, dashed down the stairs, passed the gate of the hospital, and never ceased running until he reached his cabin in Pallas-Kenry, a distance of about twelve miles. He flung himself on the bed immediately, fell into a sound sleep, and awoke in the morning free from illness." (P. 211.)

Our readers, if they are as much pleased with this work as we are, will be glad to find that we have not yet come to an end, and that, although Chap. ix. was headed "Concluding Observations," we still have some more last words, in the shape of Chap. x. on *Treatment*.

"The following distinction of cases," says our author, "which differs little from that given by Dr. Brown, of Glasgow, will be found to answer all useful purposes in practice.

"Cases of pain affecting a single nerve, with tenderness at a corresponding part of the spinal column, and little or no constitutional disturbance.

"Cases of a more complex nature, with tenderness of the spinal column to a greater extent, and continued symptoms of disorder in the digestive or uterine, or sometimes in the cerebral functions.

"Cases of a similar description, but in which the disturbance of function in the different organs appears subsequently to other manifestations of the disease, or exist evidently as a secondary affection. These are chiefly the instances in which we observe a frequent metastasis of the diseased action from one set of organs to another." (P. 223.)

In the first class, the chief remedies are blistering and leeching the tender spot, and the internal use of narcotics, such as belladonna, or tonics, particularly the carbonate of iron and sulphate of quinine. Those who rely on local remedies alone will often be disappointed. Gastrodynia, when it seems to depend on the presence of acrid juices, or a morbid sensibility of the mucous membrane, is sometimes benefited by the use of the carbonate of iron, and compound powder of jalap. The oxide of bismuth, small doses of opium and kino, and the acetate of lead, given, as Mr. Gardiner recommends, with dilute acetic acid, in doses of two or three grains three times a day, have all sometimes been advantageous.

" But all these remedies have seemed very inferior in efficacy to a popular one among the poor in this country, which we have fallen upon by accident, the super-sulphate of alumen. We first saw it used in the case of a patient afflicted with pain of stomach, sometimes occurring in violent paroxysms, and accompanied by vomiting and pyrosis. There was great tenderness at the pit of the stomach, and in the right hypochondrium. The complaint had subsisted long, and he had been under a variety of treatment with little benefit. It was, in fact, eventually supposed to depend on serious organic disease of the liver and of the stomach. About this time, however, he was prevailed on, by a friend of his, to take an ounce of alum in a dose. It acted as a purgative, and gave such immediate relief, that he was induced to repeat it. The benefit he again experienced was very considerable; and, by persevering in the remedy, a cure was eventually effected. He has since, at long intervals, had a disposition to a return of the complaint, but not to any distressing degree. Latterly, he has been in the habit of substituting for the alum, half a wineglassful of vinegar when threatened with an attack, and with equal success. This latter is also a popular remedy; but probably his chief reason for resorting to it, was the disagreeableness and difficulty of swallowing such large doses of alum.

" We some time afterwards met with another case, in which the alum effected a cure. A woman, who had been long suffering with pain of stomach and pyrosis, took a tablespoonful of it powdered and mixed with sugar, twice a-day. She made use of it only two or three days, when she had the greatest relief, and had no return of the attack for months. We believe a tablespoonful is the dose usually prescribed among the poor; but so large a quantity, if generally ordered, would, we should imagine, be occasionally attended with unpleasant effects. Fortunately, it is effective in much smaller doses. We have been in the habit of prescribing it in the proportion of a teaspoonful of the powder twice a day, with two aloetic pills every night, and have been perfectly astonished at the great relief it has given; acting in some instances like a charm on a state of disorder which has resisted other remedies for years. It is, however, perfectly useless in the very minute doses in which it is commonly prescribed.

"The success with which these and other similar medicines are occasionally exhibited in cases of gastrodynia and pyrosis, accompanied by tenderness in the epigastrium, gives us a tolerable assurance that they are not always, nor even commonly, dependent on any inflammatory state of the mucous membrane. They are evidently connected with disordered functions of the nerves, which, though sometimes arising from a cause acting within the stomach itself, is more frequently attributable to irritation at the dorsal portion of the spinal cord." (P. 228.)

Dr. Griffin does not seem to make any distinction in the treatment of the second and third class; his remarks apply to both. Purgatives, leeches, blisters, and narcotics, are the chief remedies. The extract of belladonna mixed with soap plaster, is a good external application. Friction along the spine is frequently useful; issues are rarely so, and sometimes keep up the irritation they are intended to remove. We recollect that Sir Henry Halford mentions, in his Essays, a young lady, in whom epilepsy was caused by an issue. Dr. Darwin, who was consulted in the case, with the practical tact of a first-rate physician, found out the cause, eliminated the pea, and cured the case, literally, at a blow.

The recumbent posture is rarely beneficial, and often absolutely injurious, but is sometimes rendered necessary for a few hours in the day, by acute pain in the side or stomach. But reclination, however necessary, must never be constant; gentle exercise should gradually be resumed, for the morbid sensibility is kept up by perfect rest.

We have thus conducted our readers through each chapter of this admirable treatise; and we have no doubt that the ample extracts we have given will enable them to form a just opinion of its merits, and that they will coincide with us in considering it a book of great practical value. It is true that the diagnosis as well as the treatment of these spinal affections is very simple; but so are all the best points in medicine. How easy is the diagnosis of an ague, how obvious the sulphate of quinine! That is to say, when industry and talent have shown the path, it is easy to follow in it.

We could almost find it in our hearts to scold the publishers for the shabby guise in which they have brought out this book: do they not know that

Gratior est virtus veniens in corpore pulchro?

They will tell us in reply, that good wine needs no bush; and we must confess that a more sparkling or invigorating draught has rarely been presented to us.

